# Supplementary material for: SUMO-Activating Enzyme Subunit 1 (SAE1) Is a Promising Diagnostic Cancer Metabolism Biomarker of Hepatocellular Carcinoma
Source: Cells. 2021 Jan 17;10(1):178. doi: 10.3390/cells10010178 (PMC7830456; doi:10.3390/cells10010178)
Supplement: Supplementary file 1 [file cells-10-00178-s001.pdf]

SUPPLEMENTARY INFORMATION

SUMO-Activating Enzyme Subunit 1 (SAE1) is a Promising Diagnostic Cancer Metabolism Biomarker of Hepatocellular Carcinoma

Jiann Ruey Ong[1,2,3#], Oluwaseun Adebayo Bamodu[4#], Nguyen Viet Khang[4], Yen-Kuang Lin[4], Chi-Tai Yeh[4,5], Wei-Hwa Lee[6] and Yih-Giun Cherng[7,8*]

1 Department of Emergency Medicine, Shuang-Ho Hospital-Taipei Medical University, New Taipei City, Taiwan.

2 Graduate institute of injury prevention and control, Taipei Medical University, Taipei, Taiwan.

3 Department of Emergency Medicine, School of Medicine, Taipei Medical University, Taipei, Taiwan.

4 Department of Medical Research & Education, Taipei Medical University - Shuang Ho Hospital, New Taipei City, 235, Taiwan.

5 Department of Medical Laboratory Science and Biotechnology, Yuanpei University of Medical Technology, Hsinchu 300, Taiwan.

6 Department of Pathology, Taipei Medical University-Shuang Ho Hospital, New Taipei City, Taiwan

7 Department of Anesthesiology, Shuang Ho Hospital, Taipei Medical University, New Taipei City, Taiwan.

8 Department of Anesthesiology, School of Medicine, College of Medicine, Taipei Medical University, Taipei, Taiwan.

# These authors have contributed equally to this work

* Correspondence:

Dr. Yih-Giun Cherng, MD

Department of Anesthesiology, Shuang Ho Hospital, Taipei Medical University, New Taipei City, Taiwan. No. 291, Zhongzheng Road, Zhonghe District, New Taipei City, Taiwan 235

Email: stainless@s.tmu.edu.tw

Tel.: +886-2-2490088 ext. 8885

Supplementary Table S1. Specific primary antibodies of western blot in this study.

No.	Target	Dilution	Source	
1	SAE1	1:1000	#13585S	Cell Signaling
2	CDK4	1:1000	#2906	Cell Signaling
3	Cyclin B1	1:500	Sc-245	Santa Cruz
4	FOXO1	1:1000	#2880	Cell Signaling
5	KLF9	1:1000	ab227920	Abcam
6	GAPDH	1:500	Sc-47724	Santa Cruz

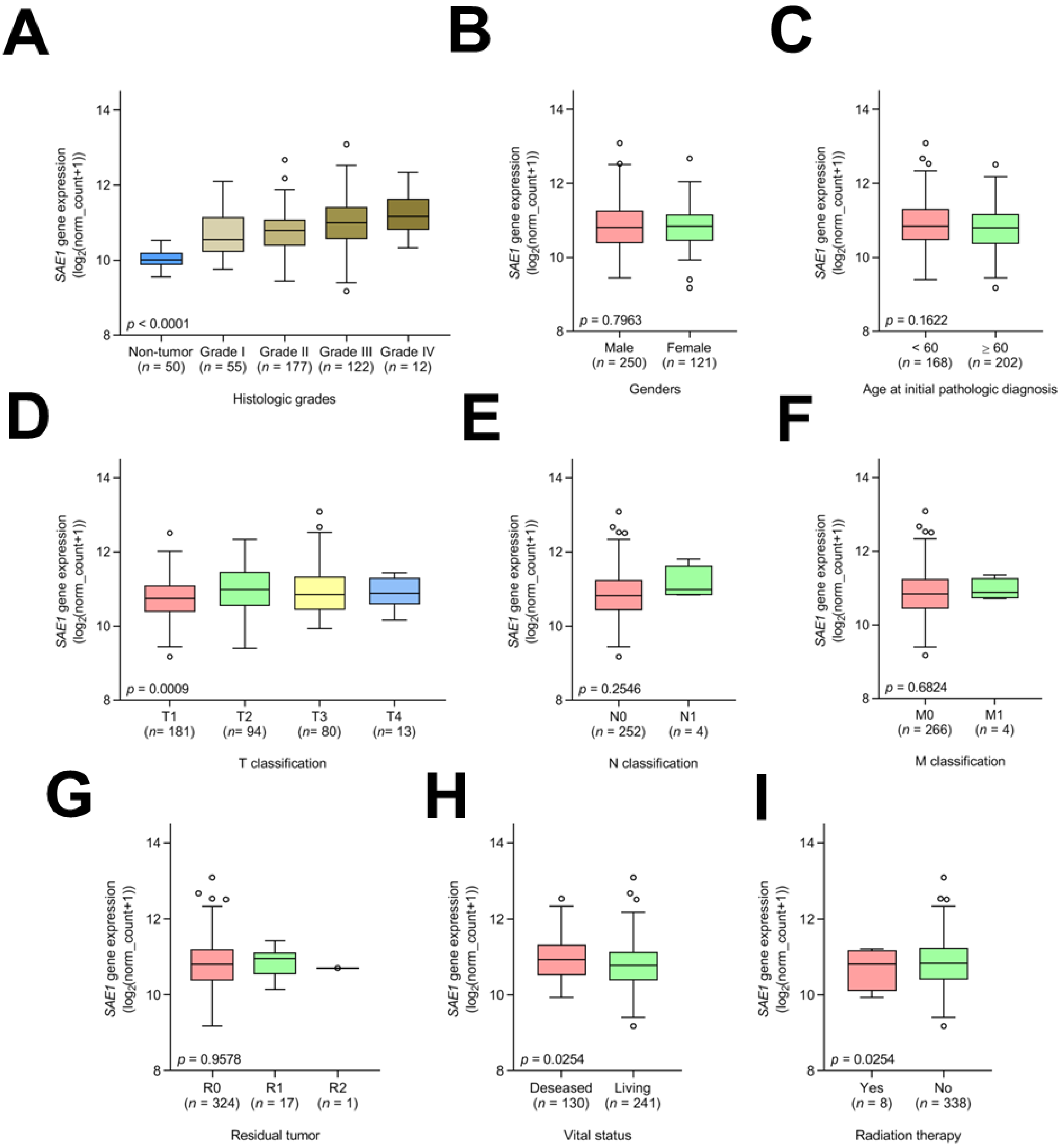

Supplementary Figure S1. SAE1 gene expression in HCC. Boxplots showing the mRNA expression levels of SAE1 according to histologic grade (A), genders (B), age at initial pathologic diagnosis (C), TNM classification (D-F), residual tumor (G), vital status (H) and radiation therapy (I).

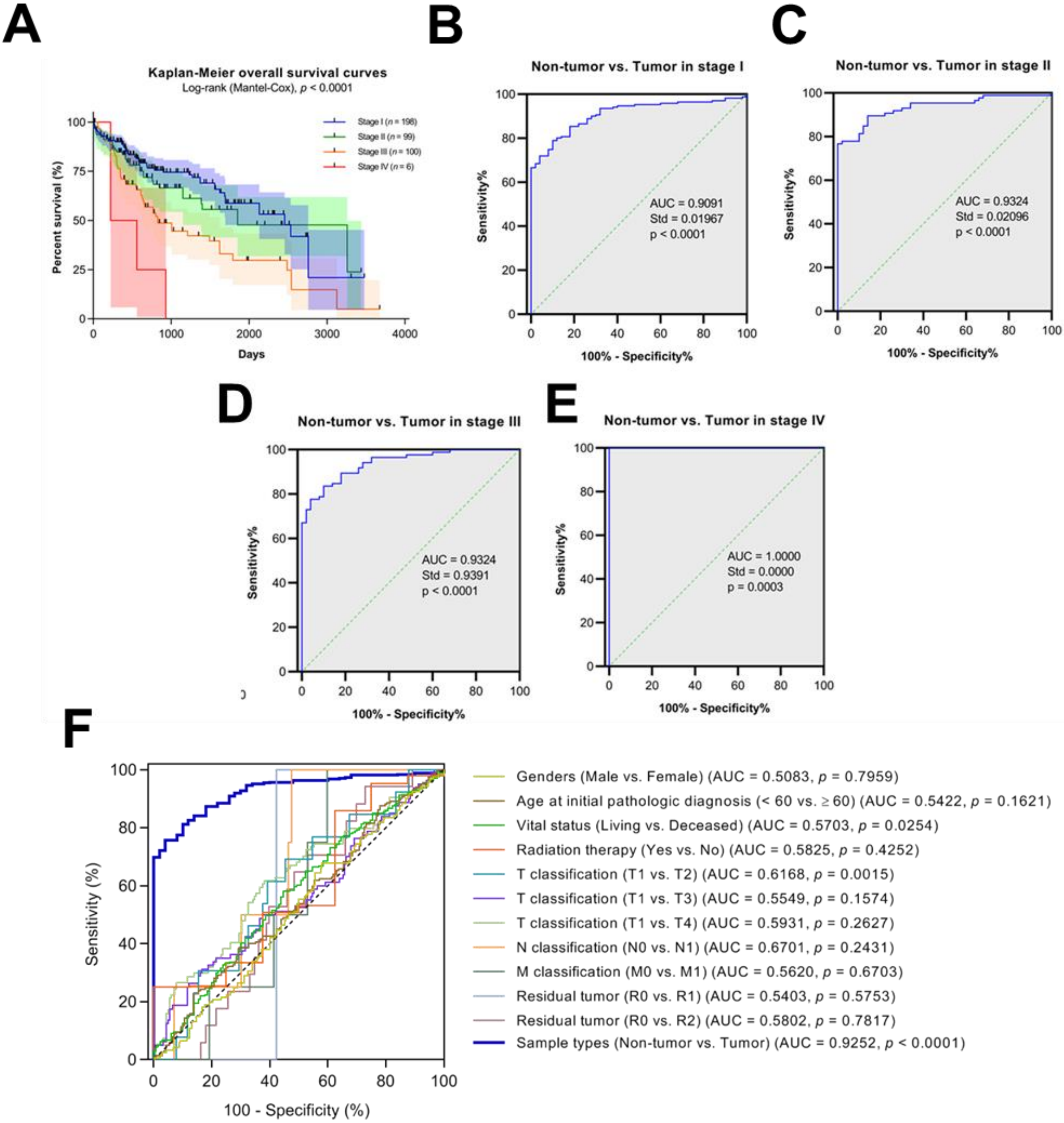

Supplementary Figure S2. Kaplan-Meier survival and ROC curves of SAE1 prognostic signature. (A) Kaplan-Meier curves of overall survival according to HCC pathologic stages. (B-E) ROC analysis of SAE1 expression in non-tumor versus tumor in stages I, II, III and IV. (F) ROC analysis of SAE1 expression characterized by various clinicopathological factors.

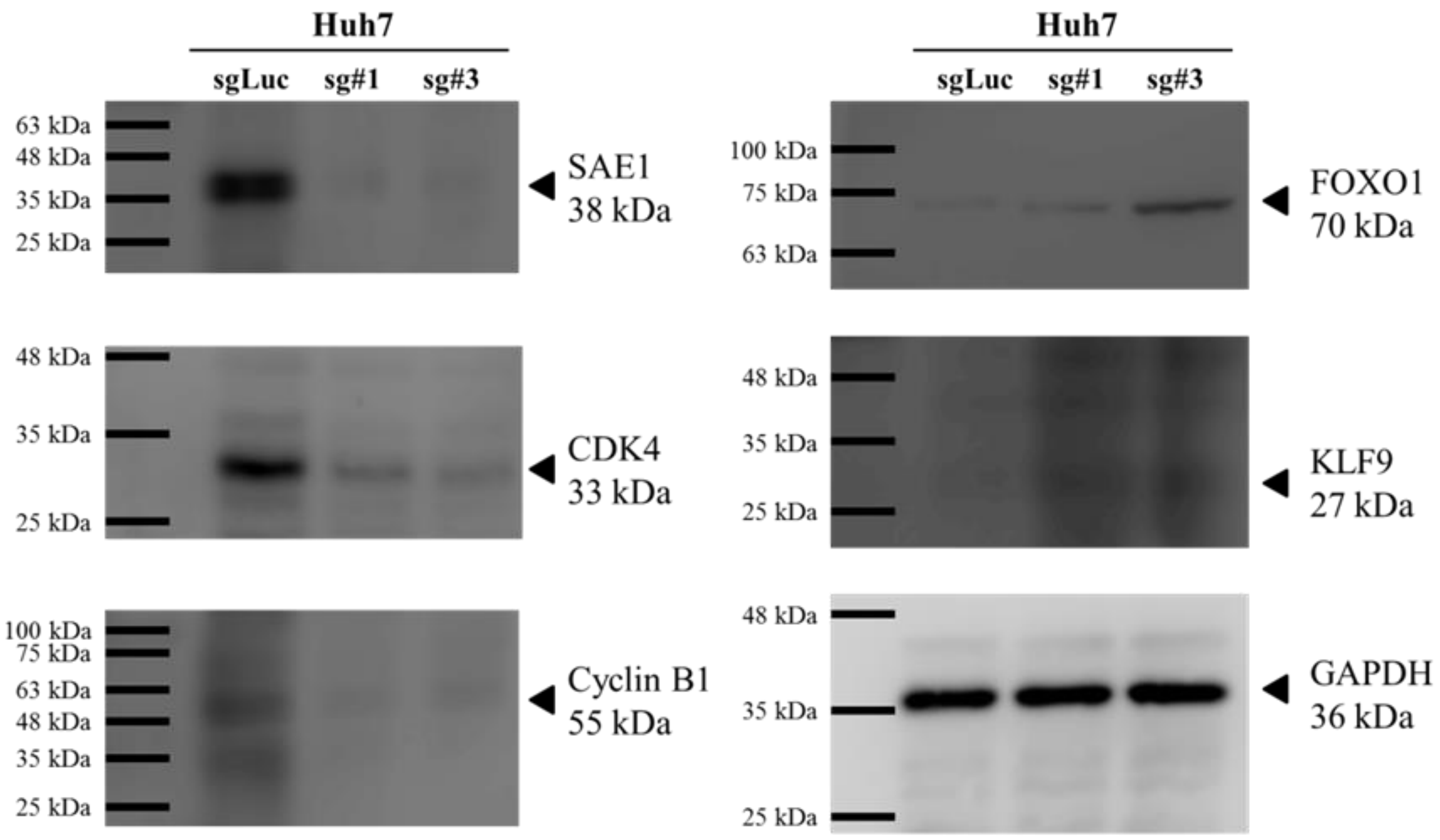

Supplementary Figure S3. Full-size blots of Figure 5E.
